# Cross-cultural perspectives on intelligent assistive technology in dementia care: comparing Israeli and German experts’ attitudes

**DOI:** 10.1186/s12910-024-01010-6

**Published:** 2024-02-07

**Authors:** Hanan AboJabel, Johannes Welsch, Silke Schicktanz

**Affiliations:** 1 Department of Medical Ethics and History of Medicine, University Medical Center Göttingen, Göttingen, Germany; 2 The Paul Baerwald School of Social Work and Social Welfare, The Hebrew University of Jerusalem, Jerusalem, Israel

**Keywords:** Empowerment, Privacy, Stigma, Cross-cultural study, Dementia, Intelligent assistive technology

## Abstract

**Background:**

Despite the great benefits of intelligent assistive technology (IAT) for dementia care – for example, the enhanced safety and increased independence of people with dementia and their caregivers – its practical adoption is still limited. The social and ethical issues pertaining to IAT in dementia care, shaped by factors such as culture, may explain these limitations. However, most studies have focused on understanding these issues within one cultural setting only. Therefore, the aim of this study was to explore and compare the attitudes of Israeli and German dementia experts toward IAT in dementia care, to contribute to a more cultural-comparative perspective.

**Methods:**

Semi-structured interviews were conducted with 35 experts (15 Israelis and 20 Germans) in key roles in health and community services for people with dementia as well as in the fields of dementia and IAT (e.g., computer science, electrical/biomedical engineering, ethics, nursing, and gerontology). Thematic content analysis was used to analyze the data.

**Findings:**

Israeli and German experts identified the same social accelerators in the development and implementation of IAT in dementia care (i.e., changes in family structure and social digitization) and benefits of adopting IAT (e.g., enhancing the safety of people with dementia and increasing their independence). However, there were differences in inhibitor/risk assessments between the two groups. Namely, economic considerations and the cognitive capacity of people with dementia were identified by both groups as inhibitors, while Israeli experts additionally reported stigma and ageism. Whereas both groups agreed that IAT might reduce human connection, and that the technology is not yet reliable enough, German experts highlighted concerns regarding privacy; in contrast, Israeli experts prioritized safety over privacy.

**Conclusions:**

Our research findings allow for the identification of relevant similarities but also important differences between German and Israeli experts’ perspectives. As such, an important basis has been provided for a more in-depth discussion regarding where, why, and how culturally-sensitive technology development is needed.

**Supplementary Information:**

The online version contains supplementary material available at 10.1186/s12910-024-01010-6.

## Introduction

For some time, scholars as well as public health care figures and the broader public have been discussing intelligent assistive technology (IAT) as an innovative tool for use in various fields of healthcare, such as dementia care. Assistive technology is a term covering a wide-ranging set of devices, products, and/or systems that can be adapted to individuals in order to improve or maintain their functional capabilities and thus enhance their independence and participation in various areas [1]. Intelligent assistive technology utilizes artificial intelligence (AI), which refers to systems that behave in an intelligent manner via an analysis of their environment and a taking of action – somewhat autonomously – to accomplish specific goals [2]. The most common IAT tools in dementia care are distributed systems (such as smart home systems), self-contained devices (such as tablets, smart phones, and wearables; e.g., GPS trackers), and humanoid robots [3]. Although studies in this regard are still in their infancy, their underlying rationale is the idea that IAT might help people with dementia to live with more independence, social connectedness, and safety [4–7], thereby empowering them [8]. Furthermore, the benefits of using IAT are not limited to people with dementia, but extend to family and professional caregivers as well. For instance, IAT improves the interactions between caregivers (both family and professional) and people with dementia, reduces worry and caregiver burden, and facilitates the work of professionals [9, 10] and, as such, enhances overall quality of care. However, IAT is often driven politically by the motivation to mitigate core challenges in today’s healthcare sector – for example, the shortage of skilled nursing staff and the ever-increasing costs of healthcare supplies [8].

Despite the above-outlined benefits and opportunities of IAT, its rate of adoption by people with dementia and their caregivers has been low [9, 11]. A main reason for this under-utilization seems to be the social and moral perceptions regarding IAT [9, 12]. Studies have shown that the use of IAT in dementia care raises various ethical challenges and dilemmas, such as concerns about the invasion of privacy, as well as the autonomy of people with dementia due to the use of surveillance technology [6, 13], concerns about the misuse of data collected via IAT, and the fear of losing control over such data [14]. Concerns about replacing human caregiving with care provided by machines are also prevalent [12, 14]. In addition, the use of IAT, especially visible technologies, may stigmatize people [15, 16].

## Sociocultural aspects and IAT

There is broad evidence that sociocultural aspects impact not only beliefs, norms, and values of individuals in a specific society [17], and perceptions about technology [18–20], but also structural dimensions of politics, such as health and technology innovation policies. For a better understanding of IAT acceptance within such a complex setting, a cultural comparison can help us gain a better understanding of such often implicit factors. Most cross-cultural studies in the field of technology have emphasized the aspect of individualism versus collectivism. Namely, a culture characterized by an individualistic approach tends to hold more positive attitudes toward technology as well as to adopt and accept new technology faster than do cultures characterized by a collectivistic approach [20–22]. These findings can perhaps be explained by one’s level of uncertainty avoidance (i.e., a dimension in Hofstede’s model that indicates the level at which a person feels in danger or threatened by unclear and incomprehensible situations) [23] – levels that tend to be higher in collectivistic societies than in individualistic societies [24–26]. Furthermore, the barriers and motivations for technology adoption may differ between the two cultures. For example, regarding barriers, people from individualistic cultures may tend to be more concerned about privacy issues (such as sharing information online) than are people from collectivistic cultures [27, 28]. As for motivations, families with a collectivistic orientation may use technology to allow old people to grow old within the family environment. By contrast, families with a more individualistic orientation may use technology to strengthen the autonomy and independence of the older person [21].

These insights reinforce the importance of conducting cross-cultural research in the context of IAT in dementia care, in particular the notion that culture also shapes health-related decisions [29] and dementia care [26]. However, the mere characterization of a culture as individualistic vs. collectivistic may not be sufficient as an explanation for IAT adoption. Rather, identifying the specific commonalities and differences between cultures may help provide a cross-cultural understanding of potential barriers to and implications of the use of IAT, thus making it possible to advance research, development, and policy, and tailor technologies to users’ needs and values. To the best of our knowledge no such cross-cultural study in this context has been conducted to date.

### Comparing Israel and Germany

Comparing Israel and Germany in the area of IAT is particularly apt. Both countries are characterized by an aging population and a very high life expectancy [30, 31], as well as public health care systems that provide high-quality and effective care for all their citizens in a relatively equal fashion [32, 33]. In addition, in both places the topic of dementia has gained much attention over the last several years. For example, in both countries special national programs to raise awareness of dementia have been created, and the issue of research and development of technology in the treatment of dementia has also received increased attention [34, 35]. However, Israel and Germany differ regarding culture. For instance, according to Hofstede’s model, German culture is characterized by a higher level of individualism, and a lower level of uncertainty avoidance, and therefore one would expect people in Germany to be less inclined than people in Israel to avoid uncertainty [23], and more inclined to accept innovation, including technological developments. By contrast, Israeli culture is characterized by a high level of collectivism, a strong orientation toward family and tradition, a high level of uncertainty avoidance, and a low level of accepting innovation, including technological developments [23]. Furthermore, when it comes to various bio-techno-ethical issues, Germany and Israel diverge from one another [29]. For example, regarding end-of-life decisions and passive euthanasia, Germany is rather permissive in comparison to Israel [29, 36]. However, Israel is more liberal regarding other bio-techno-ethical issues, for example, genetic testing and surrogacy [29, 37]. These differences have implications for older people, especially for those with cognitive decline such as individuals with dementia [26], including their perceptions of the consequences of using IAT for their care, and the reasons that might motivate or delay its use.

### The purpose of the present study

In the present study we focused on and compared the attitudes and perceptions of a variety of experts who work in the context of IAT and dementia (e.g., in the fields of computer science, electrical/biomedical engineering, ethics, nursing, and gerontology), as well as experts who hold key roles in providing services to people with dementia and their families, in Israel and Germany. Given the purpose of this study, this group is highly relevant as they have knowledge and experience in the field, and are in contact with and/or represent diverse stakeholders, such as people with dementia and their family/professional caregivers. In addition, such experts have much potential to affect public health policy.

## Method

### Participants

A purposive sampling technique was used. Multiple German and Israeli experts from a broad spectrum of relevant disciplines participated in the study. To be included in the study, participants had to belong to one of the following three groups:

A) Experts in technology engaged in research or development of IAT for older people or people with dementia/cognitive decline;
B) Experts holding key roles in health and community services for people with dementia and their families, or in the fields of healthcare politics or long-term care insurance;
C) Professionals working in associations or advocacy groups on behalf of professional caregivers of older people or people with dementia.

A total of 35 (15 Israeli, 20 German) experts participated in the study (see Table 1). Regarding the Israeli experts, nine participants were technology experts (two from the field of electrical/biomedical engineering, medicine, and gerontology, respectively, and one participant from each of the following areas: cognitive psychology, industrial design, and the field of ethics), and six participants held key roles in health and community services (three participants from non-profit organizations representing older people or people with dementia and their families, two participants from nursing management in public hospitals, and one from a welfare organization). The mean age was 56, and 73% were women. As for the German experts, nine participants were technology experts (four participants in the field of computer science, two participants in the field of nursing, and three other participants who were academic researchers in the context of human-computer interactions); nine participants held key roles in health and community services (one participant from an organization representing people with dementia and their family caregivers; one from an interest group of private nursing care providers; one from an organization that funds and organizes research in the field of healthcare; two from long-term care insurance; two from the field of healthcare politics; and two from welfare organizations); and two participants were from professional associations in the field of nursing. The mean age was 49, and 50% were women (one participant defined themselves as non-binary). Interviews were conducted until saturation of new information was achieved [38, 39], taking into consideration a determination that was made on the basis of the appropriate sample size in similar studies [40].

**Table 1:** Participants’ sociodemographic characteristics by country (*n* = 35)

	Israeli experts (*n* = 15)	German experts (*n* = 20)
**Mean age (S.D)**	55.53 (11.39)	49.0 (8.87)
**Gender (%)**		
Men	27	45
Women	73	50
Other	0	5
**Expert type (%)**		
Technology experts	60	45
Experts in key roles in health and community services	40	45
Professional association experts	0	10

### Interview guide

As is customary in qualitative research [41], we developed a semi-structured interview guide (see Appendix 1). The interview guide contained 15 questions aimed at examining the experts‘ opinions toward (1) social and technical preconditions, their experiences, and their moral perceptions regarding the use of IAT in dementia care; 2) the benefits and risks that these systems can pose to people with dementia and their family and professional caregivers; and (3) their perceptions of the optimal technical and ethical characteristics of these systems.

Pre-tests were conducted among three Israeli participants and one German participant in order to trial the interview questions and analysis method. Following pre-tests, minor corrections were made to the wording of the questions in the interview guide.

### Procedure

The interviews with Israeli experts were conducted from July 2020 to January 2022, and with German experts from July 2020 to March 2021. The Israeli and German participants were identified by their professional backgrounds as detailed in their job and academic profiles and/or via their publications on the research topic using Internet search engines (Google and Google Scholar). In addition, some of the participants were located through academic acquaintance. Participants were contacted via email or telephone. All participants signed informed consent after receiving a detailed explanation of the purpose and procedure of the study, as well as after being assured of their anonymity and confidentiality. The interviews were conducted in the mother tongue of the participants (i.e., Hebrew or German). The average length of the interviews among the Israeli participants was 35 min, and among the German participants it was 58 min. All of the Israeli and 16 of the German interviews were conducted via Zoom; in addition, three of the German interviews were conducted by phone, and one was conducted face-to-face. The reason that most of the interviews were conducted by Zoom was due to COVID-19-related restrictions that existed at the time of the interviews, including lockdowns, travel restrictions, and social distancing.

To ensure the intercoder reliability of the data, the interviews were audio-recorded and then transcribed verbatim. Unfortunately, due to budgetary and time constraints, it was not possible to translate all of the interviews into English. Therefore, researchers in each country prepared a table of detailed coding guidelines, including verbatim citations translated into English. The researchers discussed these coding guidelines until agreement was reached on a uniform coding structure (see Appendix 2). This uniform coding guided the researchers in analyzing the interviews.

### Interview analysis

The analysis of the interviews was performed using a thematic analysis approach [42]. In accordance with this approach, the data were analyzed in five stages. In the first stage, the researchers familiarized themselves with the collected data by reading the transcripts several times, after they had been transcribed verbatim. In the second stage, initial coding was conducted; at this stage, the key characteristics were coded systematically based on the uniform coding guide. In the third stage, the key characteristics constituting categories were pooled into potential themes and sub-themes, and all relevant data were collected for each category. As is customary in cross-cultural comparative studies in the field of bioethics [26, 29], we developed the categories and themes by identifying the similarities and differences in attitudes between Israeli and German experts, focusing on the ethically and culturally diverse aspects from a cross-national perspective. The categories and themes were tested and discussed by researchers from both countries until a consensus was reached. In the fourth stage, we identified and selected names for the themes: A re-examination of the themes was carried out, leading to the creation of a definition and a clear name for each theme. In the last stage, a final analysis of the data was performed by selecting compelling quotations that illustrated the theme.

## Findings

The analysis of the interviews among the Israeli (I) and German (G) experts raised three main themes: (1) the social and technical preconditions that impact IAT development and implementation; (2) the benefits of adopting IAT, and; (3) the risks of adopting IAT (see Table 2).

**Table 2:** Summary of the main themes amtong Israeli (*n* = 15) and German (*n* = 20) experts*

**1. The social and technical preconditions that impact the development and implementation of IAT** 1.1 Accelerators 1.1.1 Changes in family structure · Nuclear family units · Lack of time for family members · Less of a moral obligation to take care of one’s parents due to the strengthening of individualistic attitudes (emphasized by Israeli experts) · An increase in the number of people with dementia 1.1.2 Societal digitization · More willing to experiment with technology 1.2 Inhibitors 1.2.1. Economic considerations · The high price of existing technologies · Lack of funding sources 1.2.2 Cognitive capacity of the person with dementia · Difficulty in using IAT · Difficulty in evaluating the effectiveness of IAT 1.2.3 Ageism and stigma (emphasized by Israeli experts) · Lack of regulation · Aesthetics of IAT
**2. Benefits of adopting IAT in dementia care** *2.1 Empowers people with dementia* · Enables “ageing in place” by improving the ability of people to live independently and autonomously *2.2 Empowers family and professional caregivers of people with dementia* · Reduces the caregiver burden and worries among family caregivers · Facilitate the work of professional caregivers
**3. The risks of adopting IAT in dementia care** 3.1 *Invading the privacy of the person with dementia* (Prioritized differently by Israeli and German experts) · Israeli experts prioritize safety over privacy · Advance care directives (Reported by Israeli experts) *3.2 Lack of human contact* · Loneliness among people with dementia 3.3 *Lack of reliability of the technology* · IAT should not be considered a replacement for human care

* If no theme or subtheme is reported as typifying one particular group of experts (i.e., Israeli experts/German experts), then the theme/subtheme is relevant to both

**Theme 1. The social and technical preconditions that impact IAT development and implementation**, including accelerators and inhibitors.

***Subtheme 1.1. Accelerators*** were changes in family structure and societal digitization and were similar among Israelis and Germans.

### Changes in family structure

According to the interviewed Israeli experts, Israeli society is a collectivistic society characterized by strong family-social ties, potentially hindering the adoption of IAT in dementia care.

*“The robots, for example, that we see from Japan. Everyone I talk to in Israel about this is shocked… They ask, Why would I want a robot to take me somewhere? Pick me up? Or what is a social robot? There is no such thing. There is no substitute [for human care]. […] Israeli culture, regardless of religion or origin, is very, very family-oriented,… Maybe we can convince some people, but I doubt it [meaning, using a robot]… Even robotic dolls, robotic animals – in my eyes they’re great, but people tell me, No. I haven’t met anyone who says, Oh that’s a great idea.”* (*I, gerontologist)*.

However, some Israeli experts claimed that this (self)-perception is changing, that Israel is becoming more individualistic, and that this individualism may, in fact, drive the adoption of technology. Israeli as well as German experts also agreed that families today tend to live as nuclear rather than extended family units. In addition, children tend to live far from their parents, and family members are busy at work. The increase in the number of working women has also led to a change in caregiving patterns. Therefore, families are looking for tools that will help them fulfill their role as caregivers, and new technologies may provide a good solution.

*“Many relatives are forced to find new ways to care for their parents due to the fact that they live and work far away from their hometown.” (G, works in a non-profit organization)*.

In addition, Israeli experts emphasized that part of the change in family structure stems from children today having less of a moral obligation to care for their parents. This change has resulted from “the individualistic approach,” which has become more prevalent in Israeli society.

*“There is an individualistic attitude, every man for himself, no commitment, and there is less commitment to others.[…] It’s unpleasant to say it, but it’s a kind of selfishness, there is less willingness to care.” (I, researcher in the field of ethics)*.

### Societal digitization

Israeli and German experts reported that technology and the digital world have become an integral part of life. This phenomenon is referred to as “societal digitization” [41]. Therefore, people today are more willing to experiment with technology, including in the context of caregiving.

*“I think that thanks to the entry of all the social media apps like WhatsApp, Facebook […] we are so accustomed to working with this technology that if there were an app designed for treatment, caregivers would feel it was accessible and would work with it. Even the patients themselves.” (I, biomedical engineer)*.

*“I believe that on a fundamental level, technology […] has taken over a big part of our daily lives, and for the elderly generations as well, [technology] has become a part of their daily reality.” (G, works in associations and an advocacy group)*.

***Subtheme 1.2. Inhibitors*** were economic considerations and the cognitive capacity of the person with dementia (among both Israeli and German experts), and stigma and ageism (primarily among Israeli experts).

### Economic considerations

Both Israeli and German experts mentioned the high price of existing technologies and the lack of funding sources as being a main reason for the inability of families, especially those with low socioeconomic statuses, to adopt these technologies.

*“What about the cost of technology? Who will pay? You’ll develop technology that is very sophisticated but very expensive, so there will be a big difference in terms of accessibility. The rich will be able to use it, but the poor less.” (I, neurologist)*.

*“It is crucial to know who’s going to bear the costs for these expensive systems.” (G, researcher in the fields of nursing and technology)*.

### Cognitive capacity of the person with dementia

Israeli and German experts reported that the cognitive capacity of a person with dementia can serve as a major barrier to the use of technologies, especially technologies that require a change in this cohort’s daily routine, such as wearable technologies.

*“As for ‘wearable technologies,’ they do not work for people with dementia. Because they forget to wear them, or they get into the shower with them.” (I, biomedical engineer)*.

Moreover, the difficulty of communicating with people with dementia makes it difficult to evaluate the effectiveness of technology, hindering the development of research in this field.

“*It is incredibly hard to measure effects of technology among people who do not communicate.” (G, chairperson of a university’s computer sciences department)*.

Study participants claimed that this issue was related to the fact that the development of technologies does not take into account the needs/abilities of people with dementia due to the developers’ lack of experience and knowledge in the dementia field:

*“One of the main problems is that those who develop the technology are young people who do not understand anything about dementia or the treatment of aging people. There are few technological ventures that take into account the needs and capabilities of people with dementia.” (I, works in a non-profit organization)*.

### Ageism and stigma

Most of the Israeli experts and one German expert reported that stigmatized beliefs about dementia, and ageism, underlie the delay in promoting research and technological development for older people with dementia. These experts believe that society does not care enough about people with dementia. Less attention is given to the topic, less money is invested, and fewer researchers are interested in working in the field, especially given that currently dementia is an incurable disease.

*“Who sees old people at all?[…] People are willing to invest a lot of money into technologies of all kinds, but invest less in the elderly and people with dementia. What people think is: There’s no cure for the disease, so why are we developing technology, it’s a waste of money.” (I, works in a non-profit organization)*.

*“Disabled and sick people are pushed to the margins of our society.” (G, representative of a professional nursing association)*.

Perhaps this attitude explains the lack of regulation in IAT research and development regarding dementia – findings highlighted by Israelis and Germans:

*“The legislators are very backward in enacting laws.” (I, gerontologist)*.

*“If you really want to launch something, a medical device, in the end, the German regulations are very, very strict.” (G, technology developer)*.

The technologies used in dementia care are thus accompanied by stigma and negative attitudes (attitudes indicating that these technologies signal weakness and disability), making people less likely to adopt them. Therefore, it is not surprising that the Israeli experts reported that aesthetic considerations are essential if we wish to increase technology adoption. For example, one participant said:

*“The technology at home should be invisible. It should be an integral part of the home, like just another piece of furniture. That is, people should not see in the technology a reminder of old age or disability.” (I, industrial engineer)*.

**Theme 2. The benefits of adopting IAT in dementia care** included empowering people with dementia as well as family and professional caregivers (agreed upon by both the Israeli and German experts).

### Subtheme 2.1. Empowering people with dementia

Participants believed that technology has the potential to empower people with dementia by promoting their ability to “age in place.” Technology can give this population the opportunity to live in their community for a longer time, and can delay their transition to long-term care institutions.

*“There is definitely the possibility that people with dementia will be able to stay longer in their own homes and not yet have to go to a nursing home.” (G, works in a non-profit organization)*.

German participants noted that at each stage of dementia there are specific technologies that can help this cohort. The Israeli experts diverged a bit on this point; they emphasized that technology can be effective for people with dementia mostly in the early stages of the disease:

“*In the initial stage of dementia, technology has a major role in helping the person manage their life with [the disease] as independently as possible.” (I, gerontologist)*.

Both Israeli and German experts believed that technology could increase “aging in place” by maintaining or promoting the health of people with dementia, assisting them in performing daily activities indoors, and giving them the opportunity to be mobile outside the home. Regarding matters of health, participants viewed IAT (e.g., sensor-based monitoring systems) as an effective means for monitoring health dimensions and determining diagnoses in advance for various diseases. Intelligent assistive technology also allows (e.g., through certain applications) for monitoring patients’ cognitive status and detecting any deterioration.

*“Doctors can track patients’ health metrics […] They are now trying to develop apps to gather health indices from cell phones. This way you will be able to see the change in patients’ behavior, and you can follow the process of deterioration. By using these apps, you can tell whether the patient is not managing well, for example, if he inadvertently pressed the buttons on his phone more than once….” (I, neurologist)*.

In addition, sensor-based monitoring systems can increase the safety of this population inside the home by detecting dangerous/unusual behavior. For example, such systems can detect falls. Unlike with standard technology (such as a distress button), smart technology allows people not to have to press a button. The system can detect falls/distress and send an alert to family members, thus allowing for timely care and preventing a deterioration in the person’s condition.

*“There are actually opportunities to detect the need for support, or a sudden difference in the need for support, for example sensor mats that you can put at the front door or in front of the bed. They can be used to create movement profiles of the person with dementia living alone.” (G, representative of nursing administration in a federal state)*.

As for assisting them in performing daily activities indoors, participants claimed that technology can help this population perform daily activities at home independently. For example, one participant said:

*“Technology can provide reminders to people with dementia. For example, if a person with dementia goes to the refrigerator, the system will recognize that he is going to the refrigerator, and because it is smart technology, the system will recognize that it is now 10 o’clock and usually at 10 o’clock this individual [with dementia] drinks coffee. He takes out the milk for the coffee, and then the system guides him on how to make the coffee.” (I, industrial designer)*.

Finally, technology (especially tracking technologies, such as GPS) can increase this population’s autonomy and enable them to leave the house. Indeed, participants argued that using GPS is a good way of allowing people with dementia to enjoy being mobile and going outside in a safe manner:

*“I’ve seen these GPS tracking systems in action…and I see really great opportunities for outdoor mobility, because when using these technologies, social participation, security, and safety do not necessarily contradict one another.” (G, representative of public care insurance)*.

### Subtheme 2.2. Empowering family and professional caregivers of people with dementia

Israeli and German experts agreed that IAT can empower family caregivers by reducing caregiver burden. Specifically, using surveillance and monitoring systems can allow caregivers to reach their relatives remotely, thus enabling them to hold down outside jobs or otherwise lead their own lives. In addition, they argued that such technology can reduce feelings of worry and increase peace of mind.

*“For family members, technology gives them a lot of peace of mind.” (I, gerontologist)*.

“*The psychological burden is eased when you can simply sit at your desk at work and take a look at the system.” (G, representative of public care insurance)*.

In addition, the experts agreed that IAT can facilitate the work of professional caregivers by allowing them to monitor people with dementia, cut back on their work tasks, and thus enable them to be more focused on the patient’s physical and emotional care:

*“If every patient had a GPS monitoring bracelet it would give the nurses at the institutions a feeling that they have some kind of back-up, some control over the patient’s mobility.” (I, researcher in the field of ethics)*.

*“By being relieved of some tasks, as a caregiver I have more time to concentrate on more complex tasks, also for social and emotional tasks.” (G, representative of a free welfare company)*.

**Theme 3. The risks of adopting IAT in dementia care** included invading the privacy of the person with dementia (prioritized differently by German and Israeli experts), lack of human contact, and lack of technology reliability (among both Israeli and German experts).

*Subtheme 3.1. Invading the privacy of the person with dementia*: Participants described two ways in which the privacy of people with dementia is invaded: first, a breach of data security, and second, the use of monitoring and surveillance systems.

Regarding information security, in contrast to Israeli experts, German experts raised concerns about the data security of people with dementia, and the misuse of data sets by third parties (such as private care insurance or commercial companies).

“*There is a lot of worry regarding the use of public systems – the worry is that data are being used by third parties.” (G, representative of a free welfare company)*.

In addition, according to German experts, more attention should be paid to this issue:

*“The issue of data security and privacy: there is still not enough effort being made on these issues…. The question is indeed, what data do I really need?” (G, researcher in the fields of computer science and psychology)*.

As for the use of monitoring and surveillance systems, Israeli and German experts noted that technology poses a risk to this cohort’s privacy:

*“Well, there is the risk that you could be monitored in situations that you don’t want to be monitored in. You probably can‘t protect your private sphere.” (G, researcher in the fields of nursing and technology)*.

However, the Israeli experts brought up an important issue. They highlighted the fact that those who intrude on the privacy of the person with dementia are the person’s family members, and they intrude to protect the person with dementia.

*“If GPS watches are used to keep a person with dementia safe, then the infringement on his privacy is less important than his safety […]. Again, it depends on who uses it. If the children use it for their parents, then here we are talking about security.” (I, works in a non-profit organization)*.

Indeed, Israeli and German experts talked about the importance of balancing privacy and safety.

*“It is important to act in a sensitive way: balancing what is necessary and useful with what crosses the line.” (G, representative of private care insurance)*.

However, the Israeli experts stressed that the person’s safety was more important than the person’s privacy. Indeed, some of the Israeli participants argued that society puts too much emphasis on the importance of the privacy of the person with dementia. It is impossible not to intrude on the privacy of this population due to the nature of the disease, meaning that at some point they will become dependent on their caregivers to help them meet their needs.

*“It is impossible to go through dementia without there being a basic violation of everything related to a person’s privacy.” (I, works in a non-profit organization)*.

In addition, they claim that we live in a world where, in any event, privacy no longer exists:

*“Your phone knows all about you; we’re done with this nonsense [referring to privacy]. The phone knows where you were, who you talked to, what you’re looking for on the Internet […] not to mention the cameras that are everywhere.” (I, industrial designer)*.

Finally, Israeli and German experts emphasized the importance, when using these technologies, of obtaining the consent of people with dementia, assuming they can still express themselves. In addition, some of the Israeli experts suggested that the issue of whether someone wants technology to be used (at a time of life when they can no longer make such decisions) should be part of advance care directives. In this way, possible ethical dilemmas regarding privacy invasion could be avoided. The individuals’ wishes could be respected, even at a stage when they do not have the ability to express themselves.

*“I thought it might be worth advising people to think about their opinions on these technological developments while they still can. Like, when writing their wills, they could address this question: Would you like to use a GPS and/or all other such technology?”* )*I, gerontologist)*

### Subtheme 3.2. Lack of human contact

Israeli and German experts believed IAT could lead to a lack of human contact between the person with dementia and society, especially between the person and the person’s family members. For instance, family members may forgo visits with their loved ones because they can see and follow them via surveillance technologies.

*“There is a very fundamental risk regarding relationships between people: When there is a technical device involved, you no longer communicate directly.” (G, representative of a free welfare company)*.

Lack of human contact can cause an increased sense of loneliness in people with dementia. Technologies that are meant to help this population stay connected and increase their social interaction may, as such, paradoxically increase their loneliness.

*“What happens is that because I have Zoom or any other system, I will come less frequently to visit my parents. But is it enough for my parents to see me on Zoom? Or does it increase their loneliness? I think old people will say it is not a substitute. And so I think the loneliness will remain and even grow.” (I, gerontologist)*.

### Subtheme 3.3. Lack of technology reliability

Israeli and German experts indicated that system failures can occur, and that the information provided will therefore not always be accurate. They warned that technology should thus not be relied upon. Although it can be a supportive tool, it should not be considered a replacement for human care.

*“Technology is getting better and better, but at the same time we need to keep in mind that this technology is a supportive tool only and cannot be relied upon entirely, at least not at this stage.” (I, gerontologist)*.

*“We will always need a human actor to be in charge, someone … who will be in charge of the system on a fundamental level.” (G, researcher in the field of computer sciences)*.

## Discussion and conclusions

Our study’s aim was to examine and compare the experiences and attitudes of Israeli and German experts regarding IAT in dementia care. Three main themes emerged from the interviews’ analysis. First, social and technical preconditions that impact IAT development and implementation include accelerators (i.e., changes in family structure, and societal digitization) as well as inhibitors (economic considerations, the cognitive capacity of people with dementia, and stigma and ageism); second, the benefits of adopting IAT are in empowering people with dementia and their family as well as professional caregivers; and third, the most acknowledged risks of adopting IAT are the invasion of the privacy of people with dementia, the risk of reducing human connection, and the current lack of technology reliability. The study indicated quite similar perceptions among the German and Israeli experts. However, there were also some differences in perceptions and attitudes, which we would like to discuss now in more depth. These differences may have stemmed from Israel’s characterization as a more collectivistic society than Germany, according to the Hofstede model [23]. It should also be noted that Israel’s collectivistic attitude and sense of solidarity might be related to historical, societal, and cultural factors such as the Holocaust, wars, and the continuously unstable security situation [43].

First, there were differences regarding the perception of ageism and stigma as a barrier to IAT development and implementation. This perception was more pronounced among Israeli experts, and several cultural and contextual reasons might serve as an explanation for this finding. Cultural studies have shown that collectivistic societies, as Israel has often been characterized, hold more negative and stigmatizing attitudes toward people with mental illnesses or disabilities than do individualistic societies [44, 45]. The underlying reason may be that a collectivistic orientation allows less diversity; therefore, deviations from the norm are more visible or even problematized [46]. Researchers have argued that in the Israeli media, people with dementia are presented in a more neutral manner [47], whereas the German media tend to portray a more positive image [48]. Stigma is also a topic of active patient advocacy to be fought against in both countries [26], but only in Germany does there now exist a diversity of patient organizations that allow people with dementia to be active within such organizations and not just represented by family members [49]. That said, in the last decade there have been campaigns in Israel to raise the awareness of the public in general and professionals in particular regarding dementia, inter alia, reducing associated stigma [50]. Therefore, it may be that Israeli experts are more aware of stigma and its consequences than are German experts.

Privacy invasion was another area that was prioritized differently by the Israeli and German experts. Indeed, in accordance with existing concerns [8, 13, 16, 51, 52], most of the Israeli experts (11/15) and all of the Germans experts pointed to the main risk – namely, the invasion of the privacy of people with dementia via the use of surveillance and monitoring technologies. That said, privacy was most highly prioritized by German experts whereas Israeli experts prioritized safety over privacy. Indeed, former studies have shown that concerns about privacy and data protection are more highly prioritized in individualistic cultures than in collectivistic cultures [27, 28]. The importance, meaning, and implications of privacy (e.g., private information shared via information and communication technologies) may be perceived differently among different cultural groups [53]. In addition, studies show that individualistic societies give importance to “personal privacy,” whereas people from collectivistic societies place greater importance on “family privacy” and family-centered living [54]. Nevertheless, the Israeli experts did suggest using advance care directives – a form of preserving and respecting the autonomy and preferences of people with dementia [55] – as a way of addressing privacy dilemmas. In the context of the researched topic, it is essential that advance care directives also include the issue of using or not using IAT in the care of older people and the consequences of its use, including the potential invasion of privacy. The suggestion of advance care directives could thus be interpreted as a growing perception of the importance of personal privacy and respect for the wishes of people with dementia. These findings contribute to the international discussion regarding the ethical dilemmas about privacy among individuals with dementia [56]. Understanding how cultural attitudes are relevant for social acceptance is essential for developing and designing ethically robust technologies.

Finally, another crucial issue raised by our interviewed experts was the possibility of loneliness: a potential result of using tracking and monitoring technologies to replace human involvement for the sake of time and cost efficiency. However, we wish to point to the critical fact that regardless of technology or cognitive status, older people are in general at an increased risk of loneliness due to a lack of social and/or family relationships – namely, losing partners, limited social networks, and low levels of social activity [57]. These findings are particularly worrisome as loneliness is one of the risk factors for developing dementia and emotional distress and increased mortality among older people [58, 59]. In this case, IAT can be a double-edged sword. Some IATs (e.g., communication technologies) can increase social contact but lead to physical isolation, which can then lead to neglect of actual problems in everyday life and feelings of embodied loneliness. Other IATs that focus on social robots, monitoring and sensoring everyday life activities or health status, can risk limiting social contacts as caregivers feel “safe” about the well-being of people with dementia [7, 60, 61]. Hence, as IAT is not a substitute for human care it is imperative that healthcare politics ensure sufficient structures supporting social contact by professional and informal caregivers and not focus only on technology development. Overall, the topic of loneliness in this context is very important but not yet well understood and might greatly depend on the respective type and purpose of the IAT. This issue should therefore be systematically examined in IAT development.

In sum, the research findings confirm our hypothesis – namely, that culture plays an important role in shaping perceptions of IAT in dementia care. Although few differences were found between Israeli and German experts in IAT perceptions, the findings provide important insights for technology developers in terms of designing and developing IAT tailored to cultural values and preferences and for healthcare policy in terms of implementation strategies (i.e., addressing economic and other social issues such as stigma and ageism at an early point). Furthermore, formal service providers may use these findings to develop future technology-based interventions via their understanding and consideration of accelerators and inhibitors to using IAT, as well as the expected positive and negative consequences of using such IAT in dementia care. Finally, our study can help increase sensitivity among developers as well as scholars who assess and guide IAT development and implementation in terms of sociocultural aspects.

### Study limitations

Our findings should be considered in light of the study’s limitations. First, although we included a heterogeneous group of Israeli and German experts, the relatively small number of participants does not allow us to generalize from the results. However, generalizing findings is not the aim of qualitative research [62]; rather, its aim is to build working hypotheses that will allow future research to be more generalizable. Second, although we selected our experts carefully with the aim of identifying key actors with broad insights, long-held experience, and access to policy debates, our findings are limited to the perspectives of these specific experts included in the study. Also, although we aimed to have the experts from both countries be as similar as possible in their characteristics (i.e., regarding being key actors with experience and having professional roles), there were still differences between them, including sample size (15 Israelis vs. 20 Germans) and the type of profession. For example, in the Israeli context we did not manage to interview individuals who work in professional associations because the interviews were conducted at the height of the COVID-19 pandemic, and many professionals were occupied with risk management. These differences may have impacted the findings and should be considered when interpreting the results. However, samples almost always differ in main characteristics in cross-cultural studies [63], and we believe that our basic findings still provide important exploratory insights. Third, the interviews were conducted in Hebrew or German and analyzed in parallel in the respective language; that said, they were based on a joint coding guide, and similar research questions we agreed upon in English. After preparing a preliminary comparative analysis, selected quotes were translated into English. Unclear sequences were translated back and forth and critically discussed to reduce translation bias. Although selected quotes were translated into English, the translation may not have captured the nuances of the language, but as our focus was on basic attitudes, we believe the approach we used was acceptable. Finally, due to length limitations, we could not analyze and discuss all the interviews. Instead, we decided to focus on only three themes: the social and technical conditions that affect the development and implementation of IAT and other accelerators and inhibitors; the advantages of adopting IAT in dementia care; and the disadvantages of adopting IAT in dementia care. Other templates referring to, for example, the characteristics and role of IAT required in dementia care, will be published in upcoming papers.

### Electronic supplementary material

Below is the link to the electronic supplementary material.

Supplementary Material 1

## Data Availability

The datasets used and/or analyzed during the current study are available from the corresponding author on reasonable request.
