# Supplementary material for: Cross-cultural perspectives on intelligent assistive technology in dementia care: comparing Israeli and German experts’ attitudes
Source: BMC Med Ethics. 2024 Feb 7;25:15. doi: 10.1186/s12910-024-01010-6 (PMC10848426; doi:10.1186/s12910-024-01010-6)
Supplement: Supplementary file 1 — Supplementary Material 1 [file 12910_2024_1010_MOESM1_ESM.docx]

**Appendix 1: Interview Guide**

**Opening questions:**

1) Please tell us in which area of IAT you are conducting research or working.

2) To what extent does dementia care play a role in your research/work area?

3) Could you give me a brief definition of IAT in caregiving?

4) If you think about the challenges of dementia care, what can IAT contribute?

5) In your opinion, should there be any limitations to the use of IAT in dementia care?

**Key questions:**

6) Which social or technical developments make IAT necessary in dementia care?

7) Which social or technical conditions hinder the development and implementation of IAT in dementia care?

8) To what extent do politics influence research and development in this area (i.e., using IAT in dementia care) through funding and legislation?

9) To what extent do economic considerations play a role in this context?

10) To what extent do the interests of people with dementia and their caregivers (family members and professional) play a role in the development of IAT?

11) How specifically are the perspectives of people with dementia and their caregivers (family members and professional) involved in the development of IAT? In your opinion, to what extent should they be involved in this process?

12) What opportunities or risks do you see in IAT for people with dementia and their caregivers (family members and professional)?

13) In your opinion, what constitutes good IAT for dementia care both technically and ethically?

14) To what extent are you, your association, or colleagues involved in the development of IAT in dementia care? Are there opportunities to get involved in development that should be expanded? (Only for experts who hold roles in health and community services or work in advocacy groups)

**Open-ended question**

15) Is there anything else that we have not yet talked about but that you feel is necessary to mention vis a vis this topic?

**Appendix 2 : Coding guide**

| **Code** | **Definition** | **Subcode** | **Example** | **Coding rule** |
| --- | --- | --- | --- | --- |
| 1. Field of work/ research  **(Q1, Q2)** | Indication of professional activity related to IAT and dementia | - | *“Our foundation is very unique; it only works with technologies for the elderly, that's what we do all day […]. We examine technologies and startups in their various stages, and in some we invest money. In these situations we are sometimes part of the design team; for other [startups] we only give advice, like whether this device is worth the investment.” (I, gerontologist, A)* | If the expert reports several professional fields/associations/ activities, the background that is most closely related to IAT are selected. |
| 2. Definition of IAT  **)Q3)** | Formulation of a definition of IAT in caregiving | - | *“Any kind of equipment that might help with various issues such as increasing independence, preventing problems in functioning, including managerial functioning and daily functioning.” (I, biomedical engineer)* | Definitions in the narrower sense as well as cumulative mentions of examples of IAT by experts are coded. |
| 3. Inhibiting factors  **(Q7, Q8, Q9)** | Factors inhibiting the development or implementation of IAT in dementia care | 3.1 Political factors  3.2 Economic factors  3.3 Social factors  3.4 Technical factors | *“If you really want to launch something, a medical device, in the end, the German regulations are very, very strict.” (G, technology developer)*  *“What about the cost of technology? Who will pay?*  *You’ll develop technology that is very sophisticated but very expensive, so there will be a big difference in terms of accessibility. The rich will be able to use it, but the poor less.“ (I, neurologist)*  *“Who sees old people at all?[…] Who wants to deal with dementia? People are willing to invest a lot of money into technologies of all kinds, but invest less in the elderly and people with dementia. What people think is: There’s no cure for the disease, so why are we developing technology, it’s a waste of money.” (I, works in a non-profit organization B)*  *“What we also see as a problem for research and practice is the question of how to measure effects.* *It is incredibly hard to measure effects of technology among people who do not communicate.“ (G, chairperson of a university’s computer sciences department)* | Statements about political factors that may inhibit the development and implementation of IAT in dementia care are coded.  Statements about economic factors that may inhibit the development and implementation of IAT in dementia care are coded.  Statements about social factors that may inhibit the development and implementation of IAT in dementia care are coded.  Statements about technical fatcors that may inhibit the development and implementation of IAT in dementia care are coded. |
| 4. Foster factors  **(Q6)** | Factors fostering the development or implementation of IAT in the field of dementia | Social factors | *“Many relatives are forced to find new ways to care for their parents due to the fact that they live and work far away from their hometown.” (G, works in a non-profit organization)* | Statements about social factors that may foster the development and implementation of IAT in dementia care are coded. |
| 5. Characteristics of a good IAT  **(Q13)** | Formulation of criteria for a good IAT | 5.1 Technical  5.2 Ethical | *“Technology must be tailored to the person. That is, it must be tailor-made and individually tailored to each and every person. It must be adaptable because dementia progresses, age advances, clinical conditions change, and the seasons also change. That is part of the wisdom, both adapting the technology to the clinical condition of the person and adapting it to the environment." (I, industrial designer)*  *“AI is not purely data-driven and not fully autonomous, but rather is a partnership-based system, which is comprehensible and transparent, and which is not just, […]behavioral, so to speak, but which also takes into account cognitive, aspects and which is generally socio-technical and well-integrated into the socio-technical structure. That is, I do not disempower the professionals, the nursing staff; rather, I promote them, and I do so while preserving human dignity for, the people concerned.“ (G,* *technology researcher)* | Statements that formulate technical criteria that IAT for dementia care should fulfill are coded.  Statements that formulate ethical criteria that IAT for dementia care should fulfill are coded. |
| 6. Opportunities  **(Q4, Q12 (** | The opportunities that IAT can provide for people with dementia and their caregivers (Family members and professionals) | 6.1 People with dementia  6.2 Caregivers of people with dementia (Family members and professionals) | *"All the advanced technology, for example robotics, have been developed so that the person will not need assistance. This technology enables ageing in place. Ageing at home is a very significant thing; it has a lot of value. Advanced technology can and is intended for people to be able to stay at home and not have to move to an institution." (I, cognitive psychologist)*  *“The psychological burden is eased when you can simply sit at your desk at work and take a look at the system.” (G, representative of public care insurance)”* | Statements about the opportunities that IAT can provide to people with dementia are coded.  Statements about the opportunities that IAT can provide to caregivers are coded. |
| 7. The risks that IAT can pose  **(Q5, Q12)** | The risks that IAT can pose to people with dementia and their caregivers (Family members and professionals) | 7.1 People with dementia  7.2 Caregivers of people with dementia (Family members and professionals) | *“Well, there is the risk that you could be monitored in situations that you don’t want to be monitored in. You probably can‘t protect your private sphere.” (G, researcher in the fields of nursing and technology)*  *“Smart technology can definitely infringe on the privacy of foreign workers [referring to formal caregivers]. There have been complaints from foreign workers about installing cameras. It bothers them that something can watch them" (I, geriatrician).* | Statements about the risks that IAT can pose to people with dementia are coded.  Statements about the risks that IAT can pose to caregivers are coded. |
| 8. The role of people with dementia and their caregivers (Family members and professionals) in the development of IAT  **(Q10, Q11)** | Evaluation the involvement of people with dementia and their caregivers (Family members and professionals) in the development of IAT | - | *“We have a program called "XXX". As part of this program, startups can come to pilot with residents – for instance, they can do focus groups with the residents or let them experiment with the devices. The residents undertake to give feedback on these devices....” (I, gerontologist, A)* | Statements about the role of people with dementia and caregiver in the development of IAT care are coded. |
| 9. Collaboration between other stakeholders  **(Q14)** | Statement on forms of interaction with other stakeholders in the development of IAT | - | *"I would say that people are increasingly approaching the associations of those affected or professional associations, in order to get advice on development with regard to acceptance [of technology] but also user-friendliness.“ (G,* *echnology researcher )* | Statements about existing forms of interaction with other stakeholders in the area of development and use of IAT are coded. |

*Q: question; I: Israeli expert; G: German expert
